# Electrochemical Hydrogel Lithography of Calcium-Alginate Hydrogels for Cell Culture

**DOI:** 10.3390/ma9090744

**Published:** 2016-08-31

**Authors:** Fumisato Ozawa, Kosuke Ino, Hitoshi Shiku, Tomokazu Matsue

**Affiliations:** 1 Graduate School of Environmental Studies, Tohoku University, Sendai 980-8579, Japan; f-ozawa@iis.u-tokyo.ac.jp (F.O.); shiku@bioinfo.che.tohoku.ac.jp (H.S.); 2 WPI-Advanced Institute for Materials Research, Tohoku University, Sendai 980-8579, Japan

**Keywords:** electrodeposition, electrochemical hydrogel lithography, calcium-alginate hydrogel, cell culture

## Abstract

Here we propose a novel electrochemical lithography methodology for fabricating calcium-alginate hydrogels having controlled shapes. We separated the chambers for Ca^2+^ production and gel formation with alginate with a semipermeable membrane. Ca^2+^ formed in the production chamber permeated through the membrane to fabricate a gel structure on the membrane in the gel formation chamber. When the calcium-alginate hydrogels were modified with collagen, HepG2 cells proliferated on the hydrogels. These results show that electrochemical hydrogel lithography is useful for cell culture.

## 1. Introduction

Several cell culture methods have recently been developed for tissue engineering. For example, a culture surface modified with a thermo-responsive polymer has been used to collect cells in the form of sheets by reducing the temperature from 37 °C to 20 °C, and the cell sheets have been used for tissue engineering [1]. Magnetic force has been used to accumulate magnetically-labeled cells on a non-adherent surface. The accumulated cells were collected as a three-dimensional (3D) tissue organ following removal of the magnetic force [2,3]. In an electrochemical approach, alkanethiol self-assembled monolayers (SAMs) modified with RGD peptides were used to collect cells as a sheet via reductive desorption of the SAMs [4,5]. All these methods have been used to fabricate 3D tissue organs for tissue engineering.

Hydrogels have been used to provide scaffolds for tissue engineering. Calcium-alginate hydrogels are frequently used because they are formed by simply reacting alginate with Ca^2+^ in aqueous solution. Several methods have been developed for fabricating biocompatible scaffolds with special shapes from alginate hydrogels. For example, a microfluidic system has been used to mix a sodium alginate solution and a Ca^2+^ solution to fabricate spherical and linear calcium-alginate hydrogels [6]. In other reports, an alginate hydrogel without Ca^2+^ was fabricated by enzyme-induced oxidative coupling of alginates modified with phenyl groups [7]. An electrochemical method for the formation of calcium-alginate hydrogels has also been reported [8,9,10,11,12,13]. In this method, electrodes are inserted into a sodium alginate solution containing CaCO_3_ particles. H^+^ is generated near the electrode by the electrolysis of water, then the generated H^+^ reacts with the CaCO_3_ particles to release Ca^2+^ into the sodium alginate solution, resulting in deposition of calcium-alginate hydrogels on the electrode surface. In our previous study, tubular structures and microwell arrays of calcium-alginate hydrogel were constructed by electrodeposition [12,13]. However, mammalian cells on the electrodes were slightly damaged during electrochemical acidification [12,13]; in addition, carrying out electrodeposition only on the electrodes limits the applicability of the method to bioengineering.

To solve these problems, we developed an indirect method called electrochemical hydrogel lithography for the electrodeposition of calcium-alginate hydrogels. Electrochemical methods have been previously used to pattern biomaterials on solid substrates to form bionic interfaces [14,15,16]. These methods use a microelectrode to electrochemically generate reactive chemicals that cause the local detachment of species from a substrate surface. Nishizawa and coworkers named this technique “biolithography”, and demonstrated two-dimensional cell attachment and proliferation on the surface treated by biolithography [14]. In contrast, the electrochemical hydrogel lithography method described here fabricates calcium-alginate hydrogels indirectly on an arbitrary area. The present method can provide 3D hydrogels appropriate for fabricating organs on chips, since 3D hydrogels can mimic in vivo environments.

## 2. Experimental Section

**Figure 1:**
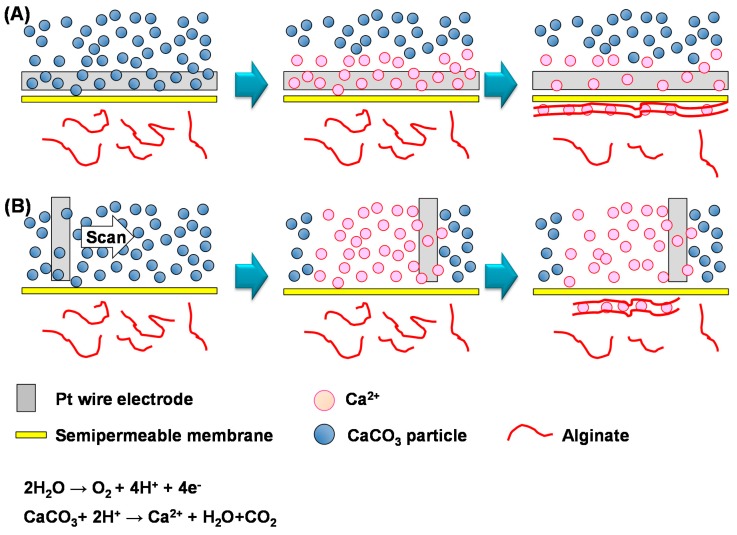
Schemes for the electrochemical hydrogel lithography of calcium-alginate hydrogels. The CaCO_3_-dispersed solution and the sodium alginate solution were introduced above (Ca^2+^ production chamber) and below (gel formation chamber) the membrane, respectively. Electrolysis of water at the Pt wire electrode produced Ca^2+^ near the electrode. The Ca^2+^ diffused to the sodium alginate solution through the membrane to form hydrogels. During electrochemical hydrogel lithography, the electrode was placed on the membrane (**A**) or scanned (**B**).

We used a semipermeable membrane to separate the chamber for producing Ca^2+^ (Ca^2+^ production chamber) by electrochemical acidification from the chamber for fabricating calcium-alginate hydrogels (gel formation chamber). This separation reduced cell damage caused by electrochemical acidification and allowed the hydrogels to be fabricated on arbitrary areas. The procedure for the electrochemical hydrogel lithography of calcium-alginate hydrogels is illustrated in Figure 1. Briefly, a 1% w/v sodium alginate solution was prepared by dissolving sodium alginate (Code No. 19-0995; Wako Pure Chemical Industries Ltd., Osaka, Japan) in a buffer solution containing 137 mM NaCl, 2.7 mM KCl, 8.5 mM Na_2_HPO_4_ and 1.5 mM KH_2_PO_4_ (PBS, pH 7.5, Wako Pure Chemical Industries Ltd., Osaka, Japan). HepG2 cells (1.0 × 10^6^ cells/mL) were suspended in the alginate sodium solution, then the HepG2 cells were cultured according to our previous paper [13]. A 1% w/v CaCO_3_-dispersed solution was prepared by dispersing CaCO_3_ in PBS. HepG2 cells (1.0 × 10^6^ cells/mL) were suspended in the above sodium alginate solution. The 1% w/v CaCO_3_-dispersed solution was placed in the Ca^2+^ production chamber, and the sodium alginate solution was added to the gel formation chamber. The two chambers were separated by a semipermeable cellulose membrane (UC24-32-100, Viskase Co. Inc., Lombard, IL, USA, MWCO: 14,000, pore size: 4–5 nm diameter, thickness: 30.5 μm) (Figure 1). The membrane prevents CaCO_3_ particles in the Ca^2+^ production chamber from passing through to the gel formation chamber, but Ca^2+^ is small enough to pass through the pores of the membrane. The membrane also prevents alginate in the gel formation chamber from passing through to the Ca^2+^ production chamber. Platinum (Pt) wire and plate electrodes were inserted into the Ca^2+^ production chamber and placed on the membrane, then a voltage of 3.1 V vs. or 4.0 V vs. the Pt plate was applied to the Pt wire electrode for 10–60 s to generate H^+^ by electrolysis of water. The CaCO_3_ particles reacted with the H^+^ near the Pt wire electrode to liberate Ca^2+^. The generated Ca^2+^ diffused to the gel formation chamber thorough the membrane and cross-linked the alginate, resulting in formation of calcium-alginate hydrogels on the membrane (Figure 1). Thus, calcium-alginate hydrogels are fabricated by indirect electrodeposition. After hydrogel formation, the membrane was washed with PBS, turned upside down, and incubated in culture medium.

## 3. Results and Discussion

**Figure 2:**
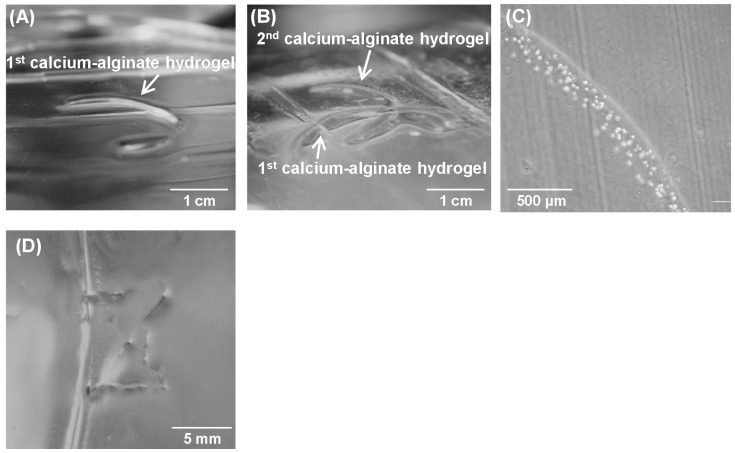
Images of calcium-alginate hydrogels obtained by placing an electrode and applying a voltage (**A**–**C**) or scanning an electrode (**D**). Image of a line-shaped hydrogel (**A**) and an overlapped line-shaped hydrogel (**B**); (**C**) Microscope image of HepG2 cells inside the hydrogel; (**D**) The Z-shaped hydrogel was fabricated by scanning the electrode.

To fabricate a line-shaped calcium-alginate hydrogel, a Pt wire electrode 200 μm in diameter was placed on the membrane (Figure 1A), then 3.1 V were applied for 60 s. The schematic illustration and picture are shown in Figure S1. Figure 2A shows that a line-shaped hydrogel was fabricated on the membrane. No hydrogel formed around the electrode because the electrode was inserted into the Ca^2+^ production chamber, which did not contain alginate. These results show that Ca^2+^ generated above the membrane diffused to the gel-forming chamber to form line-shaped hydrogels. Thus, electrochemical hydrogel lithography based on the above method is applicable to the fabrication of calcium-alginate hydrogels indirectly through the membrane. Figure 2B shows that the line-shaped hydrogels can be overlapped to generate complex patterns, indicating that a complicated pattern can be fabricated by repeated deposition. We also demonstrated that HepG2 cells can be trapped inside the deposited hydrogel (Figure 2C).

Controlled hydrogel patterns were fabricated by electrochemical hydrogel lithography using a scanning Pt wire electrode (Figure 1B). Figure 2D shows a Z-shaped hydrogel fabricated by scanning a 500-μm-diameter Pt electrode on the membrane at approximately 10 mm/min. The present method has several advantages over previous electrochemical patterning methods, including biolithography. For example, the present method can be used to fabricate 3D tissue organs in hydrogels. Furthermore, various molecules, such as drugs and growth factors, can be embedded in the hydrogels to allow the fabrication of organs on chips. In addition, since the calcium-alginate hydrogel can be decomposed by a suitable treatment, the cells can be collected after forming the tissue organs on the chip. We previously reported an electrochemical patterning method [12,13] in which hydrogel formation was limited to the electrode surface, and thus was unsuitable for fabricating flexible 3D cell-hydrogel patterns. In contrast, the present electrochemical hydrogel lithography method is a flexible tool because hydrogels can be fabricated on arbitrary areas by scanning an electrode.

**Figure 3:**
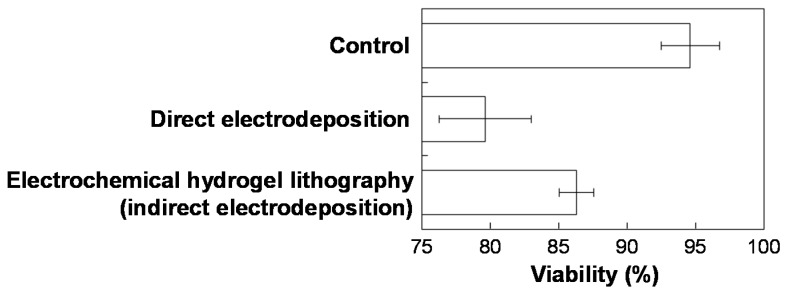
Cell viability using a conventional culture method (control), the previous electrodeposition method (Figure S2), and electrochemical hydrogel lithography (indirect electrodeposition).

After trapping HepG2 cells inside the deposited hydrogel, the calcium-alginate hydrogel was dissolved by removing Ca^2+^ from the gel using 2% EDTA in PBS, and then the cells were harvested to evaluate their viability using the trypan blue assay. Figure 3 shows improved cell viability using electrochemical hydrogel lithography (indirect electrodeposition) compared with the previous electrodeposition (direct electrodeposition) method (Figure S2). This improvement is due to the separation of electrochemical acidification (Ca^2+^-forming chamber) from the gel-forming chamber and the longer diffusion length of H^+^. However, the separation will reduce the efficiency for hydrogel formation. Cell viability using electrochemical hydrogel lithography was slightly lower compared to conventional cell culture (control), indicating that the present electrochemical deposition method still adversely affects cell culture, even though the hydrogels with cells were indirectly deposited.

**Figure 4:**
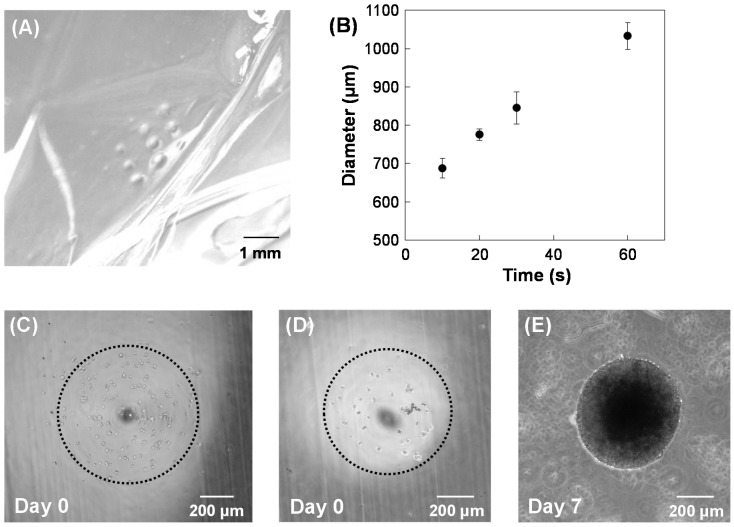
Fabrication of calcium-alginate/collagen hydrogels. (**A**) Image of a 3 × 3 dot array of the hydrogel; (**B**) Dependence of dot diameter of the hydrogels on voltage application time. HepG2 cells were embedded within the hydrogels (**C**); or seeded on the hydrogels and cultured for zero (**D**) and seven days (**E**).

Since HepG2 cells did not adhere to the calcium-alginate hydrogels or proliferate in the gels, hydrogels were modified with collagen. To modify the hydrogel, 2 mg/mL collagen type I (Nitta Gelatin, Osaka, Japan) was added to the sodium alginate solution, and then electrochemical hydrogel lithography was performed to fabricate calcium-alginate/collagen hydrogels (4.0 V for 10 s). A dot array of calcium-alginate/collagen hydrogels was fabricated on the membrane by changing the position of the 500-μm-diameter Pt wire electrode (Figure 4A). The size of the hydrogel dot was controlled by changing the voltage application time (Figure 4B). HepG2 cells (1 × 10^6^ cells/mL) were suspended in the collagen/sodium alginate solution, and then a dot pattern with HepG2 was fabricated by electrochemical hydrogel lithography. Although the HepG2 cells were successfully embedded in the calcium-alginate/collagen hydrogels (Figure 4C), the HepG2 cells did not proliferate within the hydrogels. Therefore, HepG2 cells (1 × 10^6^ cells/mL) were seeded on a dot array pattern of calcium-alginate/collagen hydrogels, incubated for 6 h, and then washed to remove unattached cells from the hydrogels. The HepG2 cells proliferated on the hydrogels after seven days of incubation (Figure 4D,E). These results suggest that calcium-alginate/collagen hydrogels can be used for tissue engineering.

Hydrogel microstructures have conventionally been constructed by optical methods [17,18]. The present method, based on electrochemical lithography, has several advantages over conventional optical methods. For example, perhaps unlike optical methods, electrochemical hydrogel lithography can use a turbid suspension of microparticles, such as magnetite nanoparticles. Furthermore, electrochemical hydrogel lithography can be applied in a space surrounded by light-shielding materials, such as in vivo. In addition, electrochemical methods can be used to analyze cells embedded in 3D hydrogels. We previously reported a large-scale integration (LSI)-based amperometric sensor consisting of 400 sensor elements for high-throughput cell analysis and bioimaging [19,20]. The electrode-array system can be easily combined with electrochemical hydrogel lithography, allowing organ construction and cell analysis on the same electrochemical chip.

Hydrogels are widely utilized in electrochemistry. Kang et al. reported a hydrogel pen using an electrochemical reaction for 3D printing [21]. By using the methodology, 3D metallic structures were successfully fabricated on the nanometer scale. In contrast, the present method can allow us to fabricate desired hydrogels. In the future, an electrochemical method will be utilized for 3D hydrogel printing.

## 4. Conclusions

We have developed an electrochemical hydrogel lithography methodology for the indirect deposition of calcium-alginate hydrogels on a semipermeable membrane. The structure and shape of the hydrogels are controllable by first placing the electrode on the membrane, and scanning the electrode. Cells incorporated in the hydrogel structure showed improved viability compared to our previous reports. Modification of the calcium-alginate hydrogel with collagen enabled proliferation of HepG2 cells on the hydrogels. These results suggest that electrochemical hydrogel lithography of calcium-alginate hydrogels is useful for culturing cells.

## Supplementary Materials

The following are available online at www.mdpi.com/1996-1944/9/9/744/s1. Figure S1: Schematic illustration and picture for electrochemical lithography. The electrode was manually positioned and scanned. For the precise control, an xyz-stage is necessary, Figure S2: Procedure for the direct electrodeposition of calcium-alginate hydrogels. Ca^2+^ is produced from the reaction between CaCO_3_ and electrolytically-generated H^+^ from water. The generated Ca^2+^ reacts with alginate directly, forming a hydrogel on the electrode. This method has been previously reported.
