# Supplementary material for: Electrochemical Hydrogel Lithography of Calcium-Alginate Hydrogels for Cell Culture
Source: Materials (Basel). 2016 Aug 31;9(9):744. doi: 10.3390/ma9090744 (PMC5457093; doi:10.3390/ma9090744)
Supplement: Supplementary file 1 [file materials-09-00744-s001.pdf]

Supplementary Materials: Electrochemical Hydrogel Lithography of Calcium-Alginate Hydrogels for Cell Culture

Fumisato Ozawa, Kosuke Ino, Hitoshi Shiku and Tomokazu Matsue

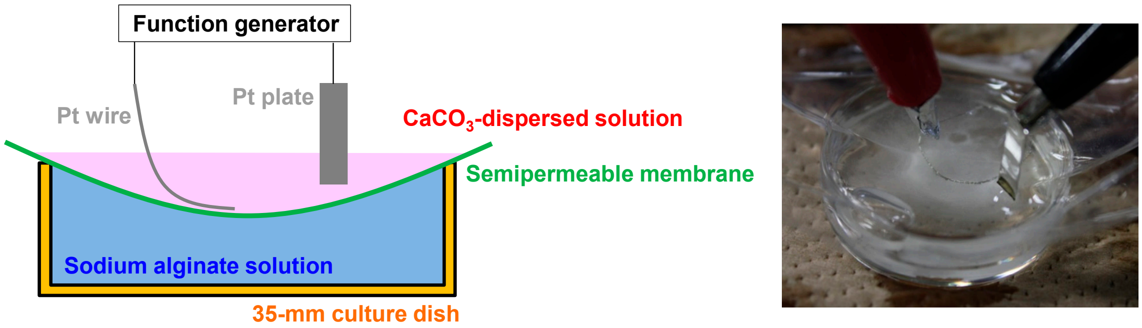

Figure S1. Schematic illustration and picture for electrochemical lithography. The electrode was manually positioned and scanned. For the precise control, an xyz-stage is necessary.

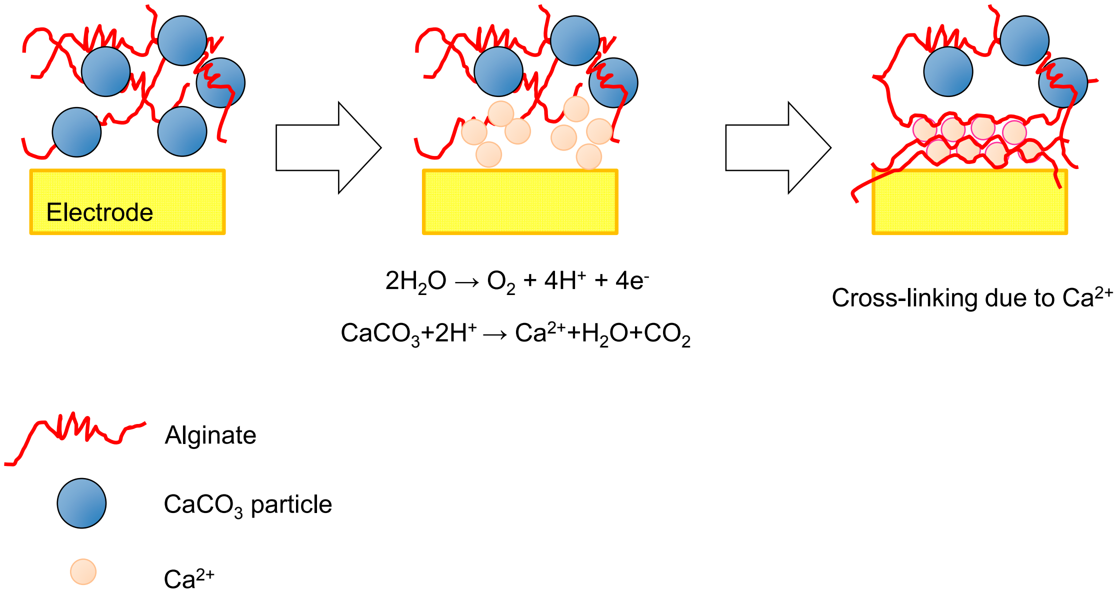

Figure S2. Procedure for the direct electrodeposition of calcium-alginate hydrogels. Ca^{2+} is produced from the reaction between $CaCO_3$ and electrolytically-generated H^+ from water. The generated Ca^{2+} reacts with alginate directly, forming a hydrogel on the electrode. This method has been previously reported [1–6].

References

1. Wang, Y.; Liu Y.; Cheng, Y.; Kim, E.; Rubloff, G.W.; Bentley, W.E.; Payne, G.F. Coupling electrodeposition with layer-by-layer assembly to address proteins within microfluidic channels. *Adv. Mater.* **2011**, *23*, 5817–5821.
2. Cheng, Y.; Luo, X.; Tsao, C.Y.; Wu, H.C.; Betz, J.; Payne, G.F.; Bentley, W.E.; Rubloff, G.W. Biocompatible multi-address 3D cell assembly in microfluidic devices using spatially programmable gel formation. *Lab Chip* **2011**, *11*, 2316–2318.
3. Shi, X.W.; Tsao, C.Y.; Yang, X.H.; Liu, Y.; Dykstra, P.; Rubloff, G.W.; Ghodssi, R.; Bentley, W.E.; Payne, G.F. Electroaddressing of cell populations by co-deposition with calcium alginate hydrogels. *Adv. Funct. Mater.* **2009**, *19*, 2074–2080.

4. Cheng, Y.; Luo, X.L.; Betz, J.; Payne, G.F.; Bentley, W.E.; Rubloff, G.W. Mechanism of anodic electrodeposition of calcium alginate. *Soft Matter* **2011**, *7*, 5677–5684.
5. Ozawa, F.; Ino, K.; Takahashi, Y.; Shiku, H.; Matsue, T. Electrodeposition of alginate gels for construction of vascular-like structures. *J. Biosci. Bioeng.* **2013**, *115*, 459–461.
6. Ozawa, F.; Ino, K.; Arai, T.; Ramón-Azcón, J.; Takahashi, Y.; Shiku, H.; Matsue, T. Alginate gel microwell arrays using electrodeposition for three-dimensional cell culture. *Lab Chip* **2013**, *13*, 3128–3135.
